# Functional Outcomes of the Knee Joint After Reconstructing the Anterior Cruciate Ligament With Peroneus Longus Tendon Graft: A Longitudinal Study

**DOI:** 10.7759/cureus.108772

**Published:** 2026-05-13

**Authors:** Satyajit Deshpande, Deepak Sanap, Vikram Sapre

**Affiliations:** 1 Orthopaedics and Trauma, N.K.P. Salve Institute of Medical Sciences and Research Center and Lata Mangeshkar Hospital, Nagpur, IND

**Keywords:** anterior cruciate ligament injuries, hamstring autograft, knee preservation surgeries, peroneus longus tendon, quadriceps tendon graft

## Abstract

Background

Anterior cruciate ligament (ACL) reconstruction is a commonly performed orthopedic procedure aimed at restoring knee stability and function. Selection of an optimal graft is crucial to achieving favorable outcomes while minimizing donor-site morbidity. The peroneus longus tendon (PLT) has emerged as a potential alternative autograft due to its adequate strength, length, and consistent diameter. This study evaluates the functional outcomes of ACL reconstruction using a PLT autograft.

Methodology

This longitudinal study was conducted at a tertiary care center between April 2023 and April 2025. A total of 23 patients with isolated ACL injuries underwent arthroscopic ACL reconstruction using a PLT autograft. Functional outcomes were assessed preoperatively and postoperatively using the International Knee Documentation Committee (IKDC) score and the Lysholm knee score at one, three, and six months. Statistical analysis was performed using SPSS version 21.0.

Results

Significant improvement was observed in both IKDC and Lysholm scores at all postoperative follow-up intervals compared to preoperative values (p < 0.05). Most patients achieved good to excellent functional outcomes by six months. No major knee-related complications were noted, and donor-site morbidity at the ankle was minimal.

Conclusions

ACL reconstruction using the PLT autograft provides satisfactory short-term functional outcomes with minimal donor-site morbidity. The PLT represents a reliable alternative graft option for ACL reconstruction.

## Introduction

The anterior cruciate ligament (ACL) originates from the medial surface of the lateral femoral condyle and attaches to the central portion of the intercondylar area of the tibia [1]. Traditionally, the ACL is recognized as the primary passive restraint against anterior translation of the tibia relative to the femur, playing a crucial role in maintaining knee joint stability [2]. ACL injuries commonly occur due to participation in sports, motor vehicle accidents, or falls. The incidence of ACL injuries among healthy athletes is estimated to be around 2.8%. The overall annual incidence of ACL tears has been reported as approximately 68.6 cases per 100,000 individuals. A study conducted in North India identified ACL injuries as the most common sports-related injury, accounting for 86.5% of cases, followed by meniscal injuries, observed in 78.24% of cases. Non-contact mechanisms were responsible for 70% of ACL injuries, while 30% resulted from direct contact [3]. Acute ACL injuries are frequently associated with concomitant injuries: approximately 5% are accompanied by lateral meniscus tears, 45% by medial meniscus tears, and 34% by medial collateral ligament injuries. These injuries often lead to acute and chronic pain, joint effusion, muscle weakness, impaired movement patterns, and reduced functional capacity. They can contribute to long-term complications such as chondral lesions and meniscal damage, significantly increasing the risk of early-onset post-traumatic osteoarthritis [4]. Furthermore, among healthy athletes, the annual incidence of primary ACL injuries is estimated to range between 1.5% and 1.7% [5].

Anterior cruciate ligament reconstruction (ACLR) is one of the most frequently performed orthopedic procedures, with over 130,000 surgeries conducted annually in the United States alone. The primary objectives of ACLR are to restore knee joint function, minimize the risk of further meniscal and chondral damage, and, ultimately, prevent the development of secondary degenerative changes within the knee [4].

Arthroscopic ACLR is widely acknowledged as a highly successful procedure, delivering consistent results for the majority of patients. Over the past couple of decades, there has been a significant evolution in the techniques of ACLR, encompassing advancements in surgical methods and a deeper understanding of biomechanics [6].

ACLR can be performed using a variety of autograft and allograft tendons. Common graft choices include bone-patellar tendon-bone (BPTB), hamstring tendon, peroneus longus tendon (PLT), quadriceps tendon autografts, as well as several allografts and synthetic grafts. Therefore, the selection of an appropriate graft is crucial for ensuring optimal long-term outcomes while minimizing donor-site morbidity [7].

Historically, the BPTB graft was widely used for ACLR. However, it carries notable disadvantages such as anterior knee pain, risk of patellar fracture, and the requirement of a more invasive harvesting technique. Additionally, the BPTB graft has a fixed length and is biomechanically weaker compared to the native ACL. In contrast, hamstring tendon grafts are easier to harvest with minimal donor-site morbidity and offer strength comparable to the native ACL. However, the diameter and size of hamstring grafts can be unpredictable, especially among the Asian population. Furthermore, harvesting hamstring tendons may result in a potential decrease in hamstring muscle strength, which could be detrimental for athletes who rely heavily on hamstring power. Complications such as infrapatellar paraesthesia, due to injury to the infrapatellar branch of the saphenous nerve, have also been reported following hamstring graft harvest [8].

One of the critical factors influencing ACLR outcomes is graft diameter, with grafts larger than 8 mm associated with reduced failure rates. However, achieving an 8 mm diameter with a quadrupled hamstring graft is often challenging. To address these concerns, orthopedic surgeons have explored the use of the PLT as an alternative graft option [9].

The PLT is located superficially on the lateral aspect of the distal leg, making it easily accessible for harvesting. Functionally, the PLT contributes to ankle plantar flexion, eversion of the sole, maintenance of the transverse arch, and stabilization of the leg on the foot. Previous studies have indicated that harvesting the PLT does not significantly impact walking ability or compromise ankle stability. Recent biomechanical evaluations have also confirmed that the strength and stiffness of the complete PLT are adequate for ACLR [10,11].

Initially evaluated through cadaveric studies, the PLT demonstrated promising results, which were subsequently confirmed in living donors. Research has highlighted several advantages of using the PLT, including good tensile strength, ease of harvest, consistent thickness, rapid regeneration at the donor site, and mechanical properties similar to the native ACL [12].

Thus, the present study was undertaken to evaluate the functional outcomes of the knee joint following ACLR using the PLT graft.

## Materials and methods

The primary aim of this longitudinal study is to evaluate knee function after the use of a PLT graft in ACLR using functional knee scores (International Knee Documentation Committee (IKDC) and Lysholm knee score) postoperatively [13,14]. We conducted a longitudinal study in the Department of Orthopaedics at a tertiary care center from April 2023 to April 2025. We included patients over 16 years of age with an ACL injury. Patients with multi-ligament injury, preexisting knee deformity/morbidity, neurovascular injury of the same limb, ipsilateral fracture of the same limb, and those with peripheral neuropathy, diabetes, and leprosy were excluded from the study. After meeting the inclusion and exclusion criteria, 23 patients were included in the study in the given time period. Prior to the initiation of the study, written informed consent was obtained from the patients. Additionally, ethical approval was also obtained from the Institutional Ethics Committee of N.K.P. Salve Institute of Medical Sciences and Research Center and Lata Mangeshkar Hospital (approval number: 62/2023).

All patients underwent preoperative assessment using the Tegner-Lysholm score and the IKDC score. In cases of acute ACL injuries, patients were advised to use a knee immobilizer and participate in preoperative physical therapy. The rehabilitation program focused on achieving near-complete knee range of motion and strengthening the knee flexor and extensor muscles to restore symmetric quadriceps strength and reduce joint effusion. Additionally, exercises aimed at activating the peroneal muscles were incorporated. Once the inflammatory phase subsided and satisfactory knee mobility was achieved, patients were scheduled for surgery.

Surgical technique

Anesthesia and Patient Positioning

All patients underwent surgery under spinal anesthesia. Patients were positioned supine on the operating table. After induction of anesthesia, ligamentous laxity was assessed using the anterior drawer test, Lachman test, posterior drawer test, and pivot shift test. A well-padded pneumatic tourniquet was applied to the proximal thigh.

Diagnostic Arthroscopy

Diagnostic arthroscopy was performed through a standard anterolateral portal. Systematic evaluation was performed in the following sequence: suprapatellar pouch and patellofemoral joint, medial gutter, medial compartment, intercondylar notch, posterolateral compartment, lateral compartment, and lateral gutter. Arthroscopic assessment confirmed a complete ACL tear in all patients. Associated meniscal or other ligamentous injuries were ruled out.

Graft Harvesting

The PLT was harvested through a vertical incision of approximately 3 cm placed just posterior to the posterior border of the distal fibula after marking the lateral malleolus. The fascia was incised carefully to expose the PLT at the posterolateral aspect of the incision. The peroneus brevis tendon was identified beneath the PLT to ensure proper differentiation. The proximal end of the PLT was secured using No. 2 Ethibond suture and transacted. The tendon was harvested proximally using a long closed tendon stripper. Care was taken to prevent peroneal nerve injury by limiting advancement of the stripper proximally and ensuring safe harvesting distance relative to the fibular head. The incision was closed using 2-0 Ethilon sutures.

Graft Preparation

After complete removal of muscle tissue, the harvested tendon length was measured. The graft was then prepared in a triple- or quadruple-strand configuration to achieve a minimum diameter of 8 mm. The final diameter was determined using cylindrical graft sizers. The tunnel diameter was selected based on the smallest sizing sleeve that allowed passage of the graft with minimal resistance. The looped end of the prepared graft was attached to an Endobutton device. The graft was pre-tensioned on a graft tension board and wrapped in gauze soaked in vancomycin solution until implantation.

Femoral Tunnel Preparation

The femoral tunnel was created using the accessory medial portal with the transportal technique. A femoral offset guide was placed at the native ACL footprint on the medial femoral condyle. With the knee hyperflexed to approximately 120°, a guide pin was advanced through the footprint. A 4.5 mm cannulated reamer was used to drill to the lateral cortex. The tunnel length was measured, and reaming was performed using a graft-sized reamer to an appropriate depth, ensuring that at least 15 mm of graft would be positioned within the femoral tunnel. An Ethibond loop was then passed through the femoral tunnel.

Tibial Tunnel Preparation

The knee was flexed to 90°. An ACL tibial guide set at 55° was positioned at the tibial ACL footprint under arthroscopic visualization. A guide pin was inserted, followed by reaming with a 4.5 mm reamer and subsequently with a graft-sized reamer. Tunnel adjustments, including medialization or posteriorization, were performed as necessary using sequential reaming.

Graft Passage and Fixation

The Ethibond loop passed through the femoral tunnel was retrieved via the tibial tunnel using a suture retriever. The graft was passed under arthroscopic visualization. The Endobutton was flipped over the lateral femoral cortex to secure femoral fixation. Cyclical tensioning was performed by flexing and extending the knee 20-30 times to eliminate creep and ensure appropriate graft tension. Arthroscopic evaluation confirmed proper graft alignment and absence of impingement. Tibial fixation was achieved using an appropriately sized interference screw.

Postoperative rehabilitation protocol

A structured rehabilitation protocol was initiated on postoperative day one. Range of motion exercises included active-assisted and active knee movements as tolerated, passive extension exercises, heel presses, prone leg hanging, and passive flexion exercises. Quadriceps strengthening included isometric contractions, straight leg raises, controlled terminal knee extension, and concentric and eccentric strengthening exercises. Hamstring strengthening was also initiated. Ankle mobilization exercises included dorsiflexion, plantarflexion, active toe movements, inversion, and eversion. Patients were mobilized with walker assistance and allowed full weight-bearing as tolerated.

Outcome measures

Functional outcomes were assessed using the IKDC subjective knee evaluation form and the Lysholm knee scoring scale preoperatively at one, three, and six months postoperatively.

Statistical analysis

Data were analyzed using SPSS version 21.0 (IBM Corp., Armonk, NY, USA). Continuous variables were expressed as means and standard deviations. Statistical significance was set at p-values <0.05. Statistical tests such as the t-test and analysis of variance (ANOVA) were used.

## Results

The age distribution of the participants showed that the majority were between 31 and 40 years old, accounting for 43.48%. Participants aged 21-30 years comprised 30.43%, while 21.74% were above 40 years old. Only one (4.35%) participant belonged to the 17-20-year age group. The mean age of the study population was 32.43 ± 8.03. Table 1 demonstrates the age distribution of the study participants.

**Table 1:** Age distribution of the study participants.

Age (years)	Number of cases	Percentage
17–20	1	4.35%
21–30	7	30.43%
31–40	10	43.48%
>40	5	21.74%
Total	23	100.00%
Mean ± SD	32.43 ± 8.03	

The gender distribution of the study participants revealed that males comprised 69.57% (n = 16) of the total sample, and females comprised 30.43% (Table 2).

**Table 2:** Gender distribution of the study participants.

Gender	Number of cases	Percentage
Female	7	30.43%
Male	16	69.57%
Total	23	100.00%

The mechanism of injury was mainly sports-related, accounting for around 52.17% (n = 12), followed by motorcycle accidents, accounting for 17.39% (n = 4), and other miscellaneous causes accounted for 30.43%.

Thigh circumference was measured 10 cm above the patella, and the mean thigh circumference on the injured limb was 31.60 ± 2.22 cm, while the contralateral (uninjured) limb measured 35.26 ± 2.06 cm. Table 3 shows a comparison of thigh circumference (10 cm above the patella) between the injured and the contralateral site. This difference was found to be statistically significant with a p-value <0.0001, indicating a reduction in muscle bulk on the injured side.

**Table 3:** Comparison of thigh circumference (10 cm above the patella) between the injured and contralateral site.

Thigh circumference (10 cm)	Contralateral site	Injury site
Mean ± SD	35.26 ± 2.06	31.60 ± 2.22
t-test	-5.80	
P-value	<0.0001	

Similarly, when thigh circumference was measured 20 cm above the patella, the mean thigh circumference on the injured limb was 41.28 ± 1.94 cm, and the contralateral (uninjured) limb showed a mean circumference of 43.18 ± 2.19 cm. Table 4 shows a comparison of thigh circumference (20 cm above the patella) between the injured and the contralateral site. This finding indicated a notable reduction in thigh muscle mass on the injured side, which could be attributed to disuse atrophy following injury and the postoperative recovery period.

**Table 4:** Comparison of thigh circumference (20 cm above the patella) between the injured and contralateral site.

Thigh circumference (20 cm)	Contralateral site	Injury site
Mean ± SD	43.18 ± 2.19	41.28 ± 1.94
t-test	-3.28	
P-value	0.002	

A longitudinal assessment of knee function using the IKDC score was performed preoperatively and at one, three, and six months of follow-up. The IKDC scores progressively improved over the follow-up period after ACLR using the PLT graft. The mean preoperative IKDC score was 41.04 ± 2.35. At one month postoperatively, the score increased to 50.98 ± 2.76. This improvement continued at the three-month follow-up, where the mean score reached 67.99 ± 4.53, and further improved to 87.00 ± 3.72 at six months. Statistical analysis using repeated-measures ANOVA showed that the change in IKDC scores over time was statistically significant, with an F-value of 788.56 and a p-value <0.0001, indicating a consistent and marked improvement in knee function during the postoperative recovery period. Table 5 shows the longitudinal assessment of knee function using the IKDC score at the preoperative and postoperative follow-ups.

**Table 5:** Longitudinal assessment of knee function using the IKDC score at preoperative and postoperative follow-ups. IKDC = International Knee Documentation Committee; ANOVA = analysis of variance

Time point	IKDC score (mean ± SD)
Preoperative	41.04 ± 2.35
1-month follow-up	50.98 ± 2.76
3-month follow-up	67.99 ± 4.53
6-month follow-up	87.00 ± 3.72
ANOVA	788.56
P-value	<0.0001

A longitudinal assessment of knee function using the Lysholm score at preoperative and postoperative follow-ups was performed. The Lysholm scores showed a gradual and significant improvement over the postoperative period in patients who underwent ACLR using the PLT graft. The mean preoperative score was 39.74 ± 3.21, reflecting poor knee function. At one month postoperatively, the score increased to 52.13 ± 5.11, indicating early recovery. By three months, the score improved further to 77.43 ± 4.80, and at six months, it reached 88.70 ± 2.74, suggesting near-normal knee function. Repeated-measures ANOVA yielded a value of 695.59 with a p-value <0.0001, indicating that the changes in Lysholm scores across the follow-up periods were highly significant and reflected consistent functional improvement in knee performance over time. Table 6 shows the longitudinal assessment of knee function using the Lysholm score at preoperative and postoperative follow-ups.

**Table 6:** Longitudinal assessment of knee function using the Lysholm score at preoperative and postoperative follow-ups. ANOVA = analysis of variance

Time point	Lysholm score (mean ± SD)
Preoperative	39.74 ± 3.21
1-month follow-up	52.13 ± 5.11
3-month follow-up	77.43 ± 4.80
6-month follow-up	88.70 ± 2.74
ANOVA	695.59
P-value	<0.0001

## Discussion

ACLR can be performed using different types of grafts, such as autografts, allografts, or synthetic options, with each demonstrating varying degrees of success. Among autografts, the hamstring tendon and BPTB grafts are the most widely accepted and commonly used. With maximum loads recorded at 2,977 N for BPTB and 4,140 N for quadrupled hamstring tendon, compared to 2,160 N for the natural ligament, these grafts have shown greater tensile strength and comparable stiffness to the native femur-ACL-tibia complex.

Despite their popularity, there is still debate regarding the optimal graft choice due to certain limitations and complications associated with these options. BPTB autografts, though frequently used, are often linked to postoperative anterior knee pain and discomfort while kneeling. Furthermore, meta-analyses have shown an increased incidence of osteoarthritis following ACLR with BPTB grafts. Hamstring tendon autografts, on the other hand, may cause electromechanical delay and weakness in knee flexors. As the hamstring muscles help protect the reconstructed ACL from anterior drawer forces generated by the quadriceps, harvesting the hamstring tendon can potentially hinder postoperative rehabilitation and impair active knee flexion.

Other less conventional autograft options include the PLT, quadriceps tendon, and fascia lata. Among these, the use of the PLT autograft has recently emerged as a promising alternative to traditional grafts in ACLR. In the present study, 23 participants underwent ACLR using the PLT graft. The mean age of the study population was 32.43 ± 8.03 years. Comparable age distributions have been reported in previous studies. Rajani et al. [15] observed a mean age of 26.22 ± 5.76 years (range = 17-39 years) in their cohort of 113 patients. Ambulgekar et al. [16] reported a mean age of 34.67 ± 8.54 years in the semitendinosus group and 33.13 ± 8.59 years in the PLT group. In the present study, the gender distribution showed a clear male predominance, with 69.57% (n = 16) of participants being male and 30.43% (n = 7) being female. Similar findings have been documented in previous research. Reddy et al. [17] reported that in their cohort of 50 patients (25 in the hamstring tendon graft group and 25 in the PLT graft group), males predominated in both groups, comprising 72% in the hamstring tendon group and 80% in the PLT group. Ambulgekar et al. [16] also found a male-dominant pattern, with 28 males and 2 females in total, evenly distributed across both groups (93.33% males and 6.67% females in each). The consistency of these findings across multiple studies suggests that ACL injuries are more prevalent among males, likely due to higher participation in physically demanding activities, contact sports, and occupational tasks that involve a greater risk of knee trauma. In our study, the mechanism of injury analysis revealed that sports-related trauma was the predominant cause, accounting for 52.17% (n = 12) of cases. Motorcycle accidents contributed to 17.39% (n = 4), while other causes comprised 30.43% (n = 7) of the injuries. Nair et al. [18] documented road traffic accidents (RTAs) as the most common mode of injury (55%, n = 66), followed by sports-related injuries (37%, n = 44), with right knee involvement in 55% of cases. Hassan et al. [19] found sports injuries in 50% of cases (n = 33), RTAs in 22% (n = 14), and falls from height in 28% (n = 18). They also noted that 45% of injuries were due to contact trauma, 50% to non-contact trauma, and 5% to hyperextension. In our study, measurements taken 10 cm above the patella showed that the mean thigh circumference on the injured limb was 31.60 ± 2.22 cm and 35.26 ± 2.06 cm on the contralateral (uninjured) limb. Similarly, at 20 cm above the patella, the mean thigh circumference was 41.28 ± 1.94 cm on the injured side and 43.18 ± 2.19 cm on the uninjured side. The difference was also statistically significant (t = -3.28, p = 0.002), reflecting a considerable loss of thigh muscle mass. This reduction is likely due to disuse atrophy resulting from the initial injury and the subsequent postoperative recovery phase. In our study, the IKDC scores showed a steady and significant improvement over the postoperative follow-up period following ACLR with the PLT graft. The mean preoperative IKDC score was 41.04 ± 2.35, which improved to 50.98 ± 2.76 at one month postoperatively. By three months, the score had risen further to 67.99 ± 4.53, and at six months, it reached 87.00 ± 3.72. Repeated-measures ANOVA revealed that these changes over time were statistically significant (F = 788.56, p < 0.0001), indicating consistent and marked recovery in knee function during rehabilitation.

Similarly, Agrawal et al. [20] observed that, preoperatively, none of their patients had normal or nearly normal IKDC scores, with 40% classified as abnormal and 60% as severely abnormal. At six months postoperatively, 33.3% had normal scores, and 26.7% were nearly normal, with a reduction in abnormal (26.7%) and severely abnormal (13.3%) categories. By 12 months, 46.7% achieved normal scores, 33.3% nearly normal, and no patients remained severely abnormal, reflecting a clear trend of functional recovery. Agrawal et al. [21] in their study also found equivalent functional outcomes between PLT and hamstring tendon grafts. At one year, the mean Lysholm knee score was 99.15 in the PLT group and 99.85 in the hamstring tendon group, while the mean IKDC score was 94.13 and 95.12, respectively. Overall, the present study’s findings align with existing literature, showing significant improvement in IKDC scores over time after ACLR with PLT. In the present study, the Lysholm knee scores demonstrated a steady and statistically significant improvement throughout the postoperative follow-up in patients undergoing ACLR with the PLT graft. The mean preoperative score was 39.74 ± 3.21, indicative of poor knee function. At one month postoperatively, the score improved to 52.13 ± 5.11, reflecting early functional recovery. Statistical analysis using repeated-measures ANOVA yielded a value of 695.59 with a p-value <0.0001, confirming that the improvements observed across the follow-up intervals were highly significant and consistent, reflecting progressive functional restoration. Sheikh et al. [22] reported that preoperative Lysholm scores were poor in the majority of cases (85%), but by nine months postoperatively, 95% of patients achieved good or excellent outcomes. Further, Rhatomy et al. [23] also found no significant difference in Lysholm and IKDC scores between different graft groups, supporting the reliability of the PLT as an effective graft choice.

Limitations

The limitations of this study include a small sample size, short-term follow-up, and a lack of a comparison group using other graft types.

## Conclusions

ACLR remains the gold standard for restoring knee stability and function following ACL injury, with graft selection playing a pivotal role in determining surgical success and patient outcomes. The findings of this longitudinal study demonstrate that the use of the PLT autograft yields significant and consistent improvements in knee function, as evidenced by progressive increases in IKDC and Lysholm scores over the six-month follow-up period. The PLT autograft proved to be a reliable graft option, offering adequate graft diameter, favorable biomechanical strength, and ease of harvesting. Importantly, it was associated with minimal donor-site morbidity, with no significant functional compromise observed at the ankle. These advantages address some of the limitations of traditional graft choices, such as hamstring and BPTB grafts. Overall, the study supports the PLT as a safe, effective, and viable alternative for ACLR, particularly in cases where conventional graft options may be suboptimal. However, given the study’s limitations, including a small sample size and short-term follow-up, further large-scale, randomized controlled trials with longer follow-up durations are recommended to validate these findings and establish long-term outcomes.
